# Relationship between Sum of the Four Limbs' Pulse Pressure and Brachial-Ankle Pulse Wave Velocity and Atherosclerosis Risk Factors in Chinese Adults

**DOI:** 10.1155/2015/434516

**Published:** 2015-01-28

**Authors:** Yansong Zheng, Zongbin Li, Hua Shu, Minyan Liu, Zhilai Chen, Jianhua Huang

**Affiliations:** ^1^The Chinese PLA General Hospital Health Science Center, FuXing Road 28, Beijing 100853, China; ^2^Institute of Geriatric Cardiology, General Hospital of Chinese PLA, Beijing 100853, China; ^3^Department of Geriatric Endocrinology, PLA General Hospital, Beijing 100853, China

## Abstract

The aim of the present study was to analyze the relationship between the sum of the four limbs' pulse pressure (Sum-PP) and brachial-ankle pulse wave velocity (baPWV) and atherosclerosis risk factors and evaluate the feasibility of Sum-PP in diagnosing atherosclerosis systemically. For the purpose, a cross-sectional study was conducted on the basis of medical information of 20748 adults who had a health examination in our hospital. Both Sum-PP and baPWV exhibited significant variations among different human populations grouped by gender, smoking, drinking, and age. Interestingly, Sum-PP had similar varying tendency with baPWV in different populations. And further study in different populations showed that Sum-PP was significantly positively related to baPWV. We also investigated the relationship between Sum-PP, baPWV, and cardiovascular risk factors, respectively. We found that both Sum-PP and baPWV had significant positive correlation with atherosclerosis risk factors while both of them were negatively related to HDL-c. In addition, there was a significant close correlation between Sum-PP and baPWV in the whole population (*r* = 0.4616, *P* < 0.0001). Thus, Sum-PP is closely related to baPWV and is of important value for clinical diagnosis of atherosclerosis.

## 1. Introduction

Cardiovascular diseases are the major factor causing sudden death in adults [1]. Numerous studies have indicated that pulse pressure (PP) is a strong independent predictor of cardiovascular diseases [2–4]. Elevated pulse pressure has been shown to be a late manifestation of reduced arterial elasticity, which is an important aspect of the arteriosclerosis diagnosis [3]. However, certain differences exist between blood pressure (BP) of human four limbs as well as PP [5]. Moreover, single unilateral measurement of pulse pressure could deviate from the actual value due to some pathological factors, such as arteriosclerosis and vascular malformations [6, 7]. Synchronous measurement of four limb blood pressure is the most accurate method of diagnosis of hypertension [6]. Thus, the difference of four limbs blood pressure and missed diagnosis of hypertension due to local factors can be avoided. Therefore, we propose that the sum of the four limbs' pulse pressure (Sum-PP) could give us a better understanding of the pathogenic cardiovascular systemically, such as arteriosclerosis. It has been reported that brachial-ankle pulse wave velocity (baPWV) can accurately reflect the arterial elasticity and has been commonly used as an indicator of arteriosclerosis [8, 9]. The aim of the present study was to evaluate the clinical significance of Sum-PP. To this end, we analyzed the relationship between Sum-PP, baPWV, and cardiovascular risk factors by Pearson's correlation coefficient.

## 2. Results

### 2.1. Baseline Characteristics of Subjects

The subjects involved in this study were from 30 different provinces, autonomous region, municipality, and special administrative region. The characteristics of subjects including general information (such as gender and age), laboratory analyses, and data collected by Arteriosclerosis Detector VBP-9 were shown in Table 1.

### 2.2. Sum-PP and baPWV in Different Populations

**Figure 1:**
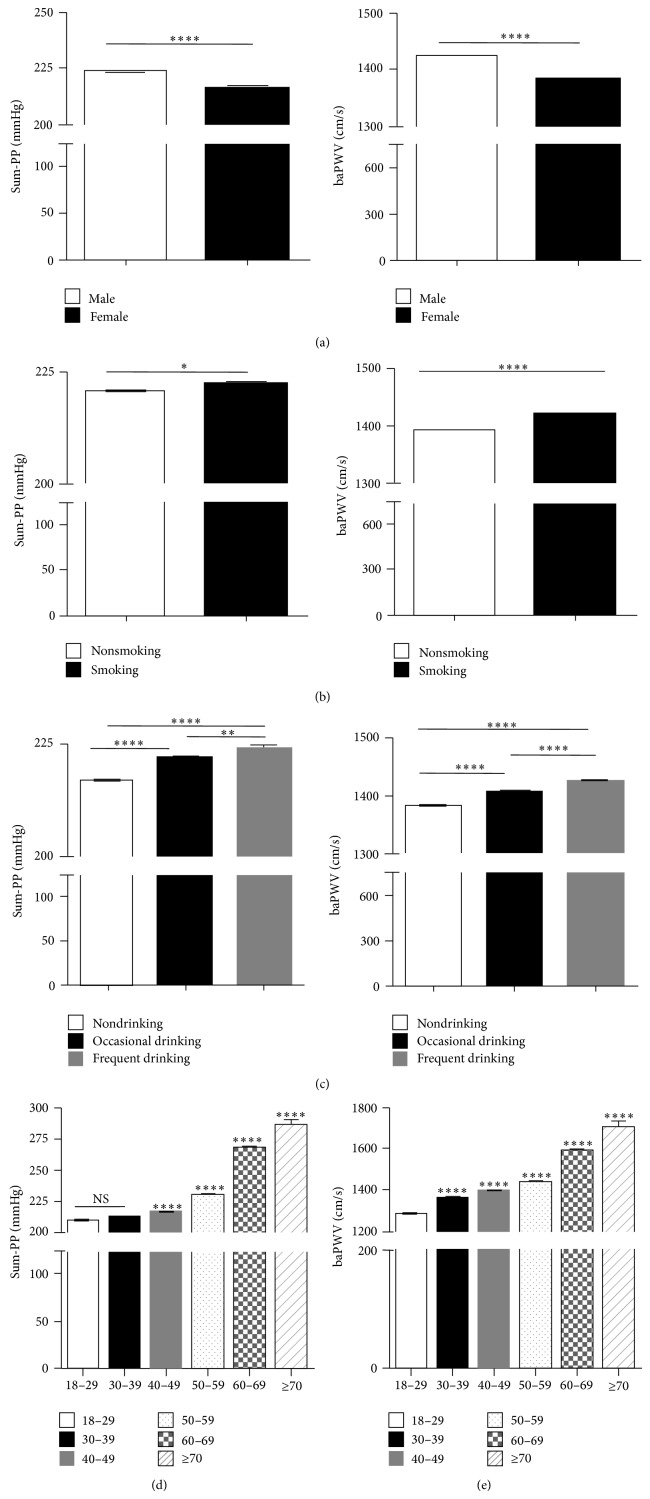
Sum-PP and baPWV have significant difference and show similar variation trend among different human populations. Sum-PP and baPWV varied with gender (a), life style (b and c), and age (d and e). The statistical significance of the differences between groups was by *t*-test. ^*^ *P* < 0.05, ^**^ *P* < 0.01, ^***^ *P* < 0.001, ^****^ *P* < 0.0001.

To investigate the correlation between Sum-PP and baPWV, we firstly analysed the variance of Sum-PP and baPWV in different populations. The subjects were divided into different population based on gender, age, cigarette smoking, and alcohol consumption. Statistical analysis showed that Sum-PP was significantly higher in male than in female (Figure 1(a), left panel). And similar varying pattern was exhibited by baPWV (Figure 1(a), right panel). Both smoking (Figure 1(b)) and drinking (Figure 1(c)) can dramatically elevate Sum-PP and baPWV. With aging, Sum-PP and baPWV were significantly increased gradually (Figures 1(d) and 1(e)). Thus, age, gender, and lifestyle do have effect on Sum-PP and baPWV.

### 2.3. The Relationship between Sum-PP and baPWV in Different Populations

**Figure 2:**
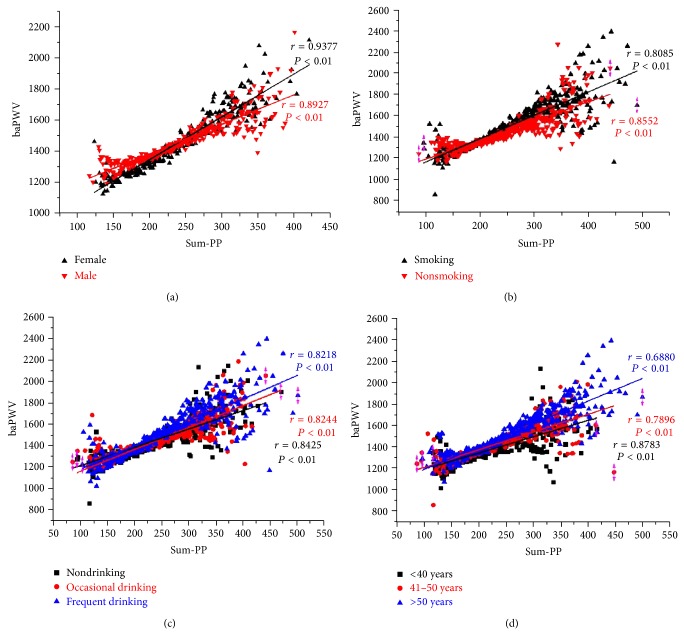
Sum-PP and baPWV show a significant correlation among different human populations. The average baPWV (a-ba-PWV) of patients with same Sum-PP was obtained. This a-ba-PWV and Sum-PP constitute a dot (Sum-PP, a-ba-PWV). The correlation between Sum-PP and baPWV was analyzed with Pearson's correlation coefficient using Origin Pro 8.

Interestingly, in the population studied above, Sum-PP always changes in the same direction with baPWV. We hypothesized that Sum-PP is related with baPWV. To this end, we did correlation analysis between Sum-PP and baPWV. As expected, Sum-PP was significantly positively related with baPWV in different populations: male (*r* = 0.8927, *P* < 0.01), female (*r* = 0.9377, *P* < 0.01); smoking (*r* = 0.8085, *P* < 0.01), nonsmoking (*r* = 0.8552, *P* < 0.01); nondrinking (*r* = 0.8425, *P* < 0.01), occasional drinking (*r* = 0.8244, *P* < 0.01), and frequent drinking (*r* = 0.8218, *P* < 0.01); <40 years (*r* = 0.8783, *P* < 0.01), 41–50 years (*r* = 0.7896, *P* < 0.01), and >50 years (*r* = 0.6880, *P* < 0.01) (Figure 2).

### 2.4. The Correlation between Sum-PP and baPWV and Atherosclerosis Risk Factors

Meanwhile, the relationship between Sum-PP and baPWV and atherosclerosis related indicators was also investigated. As Tables 2 and 3 showed, Sum-PP and baPWV were significantly related with BMI (*r* = 0.3511 and 0.1385, *P* < 0.0001), BFP (*r* = 0.1266 and 0.0155, *P* < 0.0001 and *P* < 0.0254) and atherosclerosis related indicators (*P* < 0.0001). And both of Sum-PP and baPWV had a negative correlation with HDL-C (*r* = −0.0883 and −0.0495, *P* < 0.0001).

### 2.5. The Relationship between Sum-PP and baPWV in the Cohort

**Figure 3:**
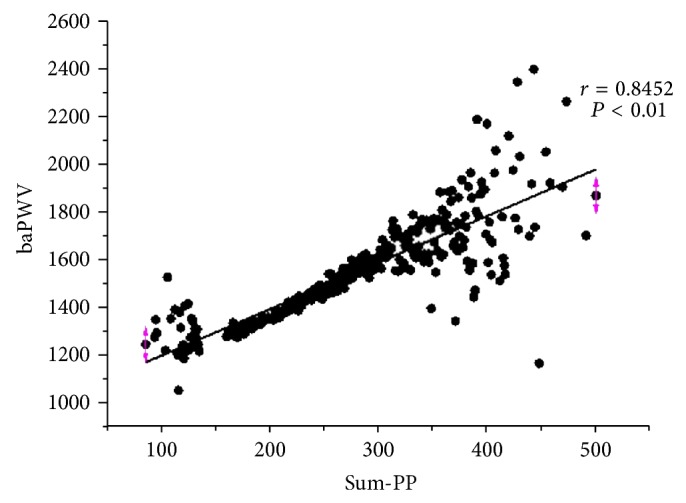
Sum-PP and baPWV were significantly correlated in the whole population. The data were processed as Figure 2. The correlation between Sum-PP and baPWV was analyzed with Pearson's correlation coefficient using Origin Pro 8.

To verify previous results, we investigated the correlation of Sum-PP and baPWV in the whole population. Again, Sum-PP was positively related with baPWV (*r* = 0.8452, *P* < 0.01) (Figure 3). Thus, Sum-PP is reliably related to baPWV, an independent predictor of atherosclerosis.

## 3. Discussion

Cardiovascular and cerebrovascular diseases are severe threats to human health. Atherosclerosis is the common basis of cardiovascular disease and cerebrovascular disease [10]. Various risk factors lead to a series of pathophysiological processes including degeneration of elastic fiber and increase of collagen fiber, which ultimately result in decreased arterial elasticity and increased stiffness, via inducing impairment of vascular endothelial function, inflammation, and hyperplasia of vascular smooth muscle [11, 12]. Pulse wave velocity (PWV) is the speed of blood pressure transmitted. It is mainly determined by the elasticity of arteries and has a well correlation with distensibility and stiffness of arteries [13]. Based on the above, PWV is an important objective indicator of atherosclerosis. Faster PWV implies worse distensibility, higher stiffness, and worse elasticity of arteries [14]. Thus, PWV can comprehensively reflect the result of accumulated damage on vascular exerted by risk factors, such as hypertension [15], hyperlipidemia [16], and hyperglycemia [17]. Many prospective studies suggest that PWV is an independent predictor of cardiovascular events and death. The measurement of baPWV is noninvasive, simple, reproducible, and easy for follow-up and evaluation [9, 14]. Therefore, baPWV is the most common used indicator of PWV.

A great deal of studies suggests that PP is a risk factor of cardiovascular and cerebrovascular diseases, especially coronary disease and heart failure [18, 19]. PP is more important than SBP and DBP in prognosis of the development of coronary disease and death in middle-aged and elderly populations [20]. It has been suggested that the control of PP should be included in the hypertension treatment guide. Increased PP could accelerate the process of atherosclerosis, and vice versa, forming a vicious circle [21]. Increased PP exerts greater shear stress on arteries, accelerates degeneration of elastic fibers and fracture, promotes the occurrence and rupture of aneurysm and intimal damage and endothelial dysfunction, and induces atherosclerosis and thromboembolic events [3, 22]. Thus, PP not only has a very close relationship with arterial stiffness, but also a reflection of accumulated damage on vessels exerted by risk factors. It is different from those random tested and unstable indicators, such as blood pressure and blood sugar. We believe that PP is of more great value in the prediction of cardiovascular diseases.

In the literature, unilateral limb PP is widely used in the clinical and its significance is confirmed by a batch of studies [7, 23]. However, there are differences between the blood vessels of individual's four limbs. And differences also exist between BP and PP of different limb, respectively. As a result, unilateral limb PP cannot fully reflect atherosclerosis throughout the body [7, 23]. Therefore, we propose the concept of Sum-PP for the first time. Compared with unilateral limb PP, Sum-PP derived from four limbs' blood pressure measured simultaneously is more accurate and more reliable. Sum-PP not only can amplify the reflection of pulse pressure amplification effect, but also is more close to the real situation of our body and indicates systemic atherosclerosis.

In conclusion, the subjects involved in this study represented the middle- and high-income population with certain healthcare consciousness. The participants were randomly from 30 different places all over China. The correlation between Sum-PP and baPWV widely recognized indicator of atherosclerosis. We found that Sum-PP and baPWV have the same trend of variability and are positively related in different human populations. In the entire cohort study, both Sum-PP and baPWV are positively related with atherosclerosis risk factors and had a significant positive correlation with each other (*r* = 0.8452, *P* < 0.01). Therefore, Sum-PP can reflect systemic atherosclerosis and combine with baPWV used in the assessment of arteriosclerosis.

## 4. Materials and Methods

The study is actually a review and statistical analysis of clinical data from routine physical examination; each participant was aware and freewill about the medical examination. All data were analyzed anonymously and thus no written consent was required. The review board/ethics committees of the Chinese PLA General Hospital approved this study.

Each participant was recruited from people who underwent a physical examination in the Chinese PLA General Hospital from May, 2009, to February, 2012. Individual information, such as smoking, drinking, and age, was collected through questionnaire. Other data were collected through physical examination. Data from questionnaire were collected by Hua Shu and Minyan Liu, while data from physical examination were collected by Yansong Zheng and Zongbin Li. All the data were analyzed by Zhilai Chen and Jianhua Huang.

### 4.1. Population

The cohort consisted of individuals (14028 males and 6720 females), age from 18 to 85 years, who underwent a physical examination in our hospital from May, 2009, to February, 2012. The exclusion criteria were as follows: (a) exclusion of repeated examination of a single person; (b) significant hypothyroidism; (c) arterial occlusive diseases; (d) severe liver, renal insufficiency, or cardiac dysfunction; (e) women with pregnancy.

### 4.2. Clinical Data Collection

The detailed information of the participants was recorded, including age, gender, and lifestyle (cigarette smoking and alcohol consumption). Smoking was classified into nonsmoking and smoking (≥10 cigarettes daily, more than 1 year). Alcohol consumption was divided into nondrinking, occasional drink (<1 times/week, more than 30 grams of alcohol for men and 15 grams for women), and regular drinking (≥1 times/week). Height and weight were measured and body mass index (BMI = weight/height 2 kg/m^2^) was calculated. Blood pressure was measured according to the Chinese Hypertension Prevention Guide (2005). Hypertension was determined based on the medical history and measured blood pressure.

### 4.3. Laboratory Analyses

Overnight-fasting venous blood was collected according to the quality control and testing standards of our hospital. Chemical analysis was conducted and levels of hemoglobin (Hb), high sensitive CRP (hs-CRP), total cholesterol (TC), triglyceride (TG), high density lipoprotein cholesterin (HDL-C), low density lipoprotein cholesterin (LDL-C), fasting blood glucose (FBG), blood uric acid (UA), serum creatinine (Cr), and homocysteine (Hcy) were measured by standard assays. Urinary albumin/creatinine (UmAlb/C) was also determined in morning urine.

### 4.4. Body Fat Percentage (BFP) Measurement

Weight, height, BMI, and BFP were measured by body composition analyzer (VIVENTE SILVER, Seoul, Korea). Settings of the analyzer are of the following frequency: 1, 5, 50, 250, and 500 Hz, and measurement range is between 100 Ω~900 Ω. The subjects are measured under fasting state wearing uniformed medical clothing and sandals.

### 4.5. Pulse Pressure (PP) and Brachial-Ankle Pulse Wave Velocity (baPWV) Measurement

All of the subjects wore uniformed medical clothing and calmed for at least 10 minutes. In the supine position, the subjects were examined by Arteriosclerosis Detector VBP-9 (Kingrich Science and Trade Company, Beijing, China). Both the brachia and ankles were wrapped by cuffs. The four cuffs were pressurized and slowly deflated synchronously and blood pressure (BP) and baPWV were determined by the pulse wave data collected for the same cardiac cycle. Single limb PP = systolic BP (SBP) − diastolic BP (DBP); Sum-PP = the sum of four limbs PP. baPWV was recorded as right and left. Since there was a significant positive correlation (*r* = 0.95, *P* < 0.001) between right and left baPWV, mean baPWV was used in the following analysis.

### 4.6. Statistics

Stata 11.0 software (STATA Corp) and OriginPro8 (OriginLab Corp., Northampton, MA, USA) were used for the statistical analysis. Kolmogorov-Smirnov methods were used for normality test. Data were represented as mean ± standard deviation. Statistical analysis was conducted by using *t*-test and Pearson's correlation coefficient. Values of *P* < 0.05 were considered to be statistical significant.

## Figures and Tables

**Table 1:** Baseline characteristics of subjects.

Characteristics	Values (*n* = 20748)
Age (y)	46.9 ± 8.41
Male	14028 (67.61%)
Female	6720 (32.39%)
Height (cm)	169.3 ± 7.94
Body weight (kg)	73.1 ± 12.69
BMI (kg/m^2^)	25.4 ± 3.36
Nonsmoking	13374 (64.46%)
Smoking	7374 (35.54%)
Nondrinking	8281 (39.91%)
Occasional drinking	4390 (21.16%)
Frequent drinking	8077 (38.93%)
BFP	28.2 ± 6.73
FBG (mmol/L)	5.92 ± 1.46
UA (mmol/L)	340.3 ± 91.4
Hb (g/L)	150.1 ± 15.4
Cr (mmol/L)	69.8 ± 13.5
Hcy (*μ*mol/L)	11.5 ± 5.03
Hs-CRP (mg/L)	0.21 ± 0.38
TG (mmol/L)	1.91 ± 1.71
TC (mmol/L)	4.96 ± 0.95
LDL-C (mmol/L)	2.88 ± 0.77
HDL-C (mmol/L)	1.25 ± 0.33
Sum-PP (mmHg)	221.5 ± 45.5
baPWV (cm/s)	1409.9 ± 224.5

Data are mean ± SD. BMI, body mass index; BFP, body fat percentage; FBG, fasting blood glucose; UA, blood uric acid; Hb, hemoglobin; Cr, serum creatinine; Hcy, homocysteine; Hs-CRP, high sensitive CRP; TG, triglyceride; TC, total cholesterol; LDL-C, low density lipoprotein cholesterin; HDL-C, high density lipoprotein cholesterin.

**Table 2:** Pearson correlation coefficients between baPVW and risk factor variables in subjects (*n* = 20748).

	Pearson's correlation coefficient	*P* value
BMI	0.1385	<0.0001
BFP	0.0155	0.0254
FBG	0.1978	<0.0001
UA	0.0899	<0.0001
Hb	0.1256	<0.0001
Cr	0.0627	<0.0001
Hcy	0.1821	<0.0001
Hs-CRP	0.0914	<0.0001
TG	0.0985	<0.0001
TC	0.1337	<0.0001
LDL-C	0.0975	<0.0001
HDL-C	−0.0495	<0.0001

Data are mean ± SD. BMI, body mass index; BFP, body fat percentage; FBG, fasting blood glucose; UA, blood uric acid; Hb, hemoglobin; Cr, serum creatinine; Hcy, homocysteine; Hs-CRP, high sensitive CRP; TG, triglyceride; TC, total cholesterol; LDL-C, low density lipoprotein cholesterin; HDL-C, high density lipoprotein cholesterin.

**Table 3:** Pearson's Correlation Coefficients between Sum-PP and risk factor variables in subjects (*n* = 20748).

	Pearson's correlation coefficient	*P* value
BMI	0.3511	<0.0001
BFP	0.1266	<0.0001
FBG	0.1907	<0.0001
UA	0.1041	<0.0001
Hb	0.0425	<0.0001
Cr	0.0486	<0.0001
Hcy	0.0955	<0.0001
Hs-CRP	0.0767	<0.0001
TG	0.0920	<0.0001
TC	0.0753	<0.0001
LDL-C	0.0434	<0.0001
HDL-C	−0.0883	<0.0001

Data are mean ± SD. BMI, body mass index; BFP, body fat percentage; FBG, fasting blood glucose; UA, blood uric acid; Hb, hemoglobin; Cr, serum creatinine; Hcy, homocysteine; Hs-CRP, high sensitive CRP; TG, triglyceride; TC, total cholesterol; LDL-C, low density lipoprotein cholesterin; HDL-C, high density lipoprotein cholesterin.
